# TNF-inhibitor drugs regulate human pathogenic Th17 cells through induction of IL-10

**DOI:** 10.1186/1479-5876-10-S3-P49

**Published:** 2012-11-28

**Authors:** Hayley G Evans, Nicola J Gullick, Gina J Walter, Urmas Roostalu, Klaus S Frederiksen, Jens G Gerwien, Andrew P Cope, Frederic Geissmann, Bruce W Kirkham, Leonie S Taams

**Affiliations:** 1 Centre for Molecular & Cellular Biology of Inflammation (CMCBI), King’s College London, UK; 2 Dept Rheumatology, King's College Hospital, London, UK; 3 Novo Nordisk A/S, Biopharmaceuticals Research Unit, Inflammation Biology, Måløv, Denmark; 4 Dept Rheumatology, Guy’s & St Thomas’ NHS Foundation Trust, London, UK

## Background

TNF-α inhibitor (TNFi) therapy has revolutionized the treatment of immune-mediated inflammatory diseases, including rheumatoid arthritis (RA). IL-17-producing CD4+ T-cells (Th17 cells) are considered important contributors to the pathogenesis of RA. Here we investigated the effects of TNFi drugs on the function and plasticity of human Th17 cells.

## Methods

The frequency of cytokine-expressing cells was assessed by flow cytometry. For functional studies, CD4+ T-cells and autologous CD14+ monocytes were co-cultured with anti-CD3 mAb in the absence or presence of different TNFi drugs. Cytokine secretion assays were used to re-sort cytokine-producing CD4+ T-cells.

## Results

*Ex vivo* analysis of patients with RA on TNFi therapy revealed an enrichment of Th17 cells in peripheral blood compared to those on disease-modifying anti-rheumatic drugs or healthy controls. However, we also found an increase in IL-10-producing CD4+ T-cells. The enrichment in IL-17+ and IL-10+ CD4+ T-cells, including IL-17+IL-10+ co-expressing CD4+ T-cells, was recapitulated *in vitro* by the addition of TNFi drugs (adalimumab, infliximab, etanercept, and certolizumab) to human monocyte/CD4+ T-cell co-cultures. IL-10 induction was independent of FcγR binding, IL-10 and CD4+CD25+ Tregs. TNFi-induced Th17 cells were functionally distinct as shown by an ability to modulate CD14+ monocytes in an IL-10-dependent manner. We report the identification of a transcription factor that is strongly associated with IL-10 expression in TNFi-induced IL-17+ CD4+ T-cells, and show that overexpression of this transcription factor drives IL-10 expression in primary CD4+ T-cells.

## Conclusions

TNFi drugs may exert their anti-inflammatory role, at least in part, by promoting Th17 plasticity through the induction of IL-10 expression in pathogenic Th17 cells.

